# 'Will anybody listen?' Parents’ views on childhood asthma care: a qualitative study

**DOI:** 10.3399/BJGPO.2024.0070

**Published:** 2024-10-02

**Authors:** Daniel Lange, Antje Lindenmeyer, Kate Warren, Shamil Haroon, Prasad Nagakumar

**Affiliations:** 1 Institute of Applied Health Research, University of Birmingham, Birmingham, UK; 2 Institute of Clinical Sciences, University of Birmingham, Birmingham, UK; 3 The Royal Wolverhampton NHS Trust, Wolverhampton, UK; 4 Birmingham Children’s Hospital, Birmingham, UK; 5 Institute of Inflammation and Ageing, University of Birmingham, United Kingdom

**Keywords:** asthma, child health, qualitative research

## Abstract

**Background:**

Asthma is the most common chronic disease in children, resulting in considerable morbidity and healthcare utilisation, especially in geographical areas with high deprivation. Parents play a pivotal role in children’s asthma management.

**Aim:**

To explore the views of parents whose children have asthma, regarding barriers and facilitators to receiving adequate asthma care.

**Design & setting:**

A qualitative study conducted in an urban, multi-ethnic setting with high socioeconomic deprivation and paediatric asthma-related hospital admissions.

**Method:**

The study used a pragmatic approach underpinned by a perspective of critical realism. Parents of children with asthma were recruited through purposive and convenience sampling, and data were collected through semi-structured interviews. Transcripts were analysed using thematic analysis, facilitated by NVivo12 software.

**Results:**

Ten parents participated in nine interviews. Six themes were identified relating to the following: (1) the establishment of a new life dynamic following a diagnosis of asthma; (2) the turbulent and drawn-out process of asthma diagnosis; (3) the roles and expectations of the partnership established between parents and healthcare services; (4) the importance of schools in asthma management; (5) sources and access to relevant information; and (6) the importance of social support networks. Parents frequently felt unsupported and misunderstood, particularly during the diagnostic process.

**Conclusion:**

Unmet parental educational and emotional needs, particularly around the time of diagnosis, were identified as a key barrier to adequate asthma management. Deeper understanding of gaps in support can instruct asthma care delivery and inform co-produced interventions, thus improving asthma outcomes in children.

## How this fits in

Asthma is the cause of considerable morbidity, unplanned hospital presentations and, unfortunately, mortality, in children in the UK each year. Parents play an important role in its management but often face challenges when caring for their children. This qualitative study adds to the literature by exploring parents’ views in a multi-ethnic, urban setting with high levels of socioeconomic deprivation. It offers insight into some of the barriers and facilitators they face when looking for support in providing adequate asthma care for their children.

## Introduction

Asthma is the most common chronic disease in children, with asthma attacks leading to 25 000 hospital admissions per year in the UK.^1–3^ Variations in childhood asthma morbidity and mortality have been linked to exposure to a host of biological, physical, and social factors.^4–6^

Parents hold primary responsibility for the management of their children’s asthma, which is a pivotal role that continues well into adolescence.^7,8^ However, they commonly face challenges, such as confusion regarding treatment, inadequate education, and parental stress, leading to poorer health outcomes for their children.^8–12^ Parents' and children’s experiences are impacted on by several driving forces, including the social and geographical setting and interactions with healthcare services.^13^ Having a child with asthma significantly impacts parents’ lives, with 69% taking time off work to look after their children, and 13% quitting their jobs.^14^

Given the far-reaching impact of childhood asthma and the complexity of factors influencing its management, comprehensive and nuanced investigation into the perspectives and experiences of those affected by it is foundational in addressing gaps in care and support provision. It is also unclear if parental education, ethnicity, lack of transport, finance, or the availability of basic asthma care in those living in deprived areas result in poor asthma outcomes.

This qualitative project aimed to explore the views of parents regarding the barriers and facilitators to receiving adequate asthma support for their children in an urban, socioeconomically disadvantaged setting with high rates of asthma-related hospital admissions. Additionally, it elicited their ideas on what interventions or pathways could better support them.

## Method

This study adopted a pragmatic approach and was underpinned by a position of critical realism, whereby data collected are viewed through the prism of the participant’s perspective and are subject to influences of the context in which it is produced. It is, therefore, well suited to eliciting and understanding lived experiences of people with unmet healthcare needs.

### Sampling and recruitment

The study setting was a multi-ethnic metropolitan borough in the UK, characterised by a predominantly urban population with high levels of socioeconomic deprivation and high asthma-related emergency hospital admissions for children and young people.^15,16^

English-speaking, local residents who were parents of at least one child aged ≤18 years with a doctor’s diagnosis of asthma were included in the study. Purposive and convenience sampling was achieved using a variety of recruitment routes, including project dissemination on local council media outlets, social media groups, and in primary and secondary schools. Shopping vouchers of a token monetary value were advertised and offered to all participants who completed an interview.

### Data collection

A topic guide was developed based on findings from an initial literature review and piloted before its use. Participants took part in virtual or in-person semi-structured interviews according to their preference. In one interview, two parents of the same child participated jointly. Interview duration averaged 40 minutes and took the format of an informal conversation. All participants were interviewed between June and September 2022 by the same male researcher, with a clinical background, who received training in qualitative research as part of a master’s degree. Digital recordings were used to transcribe interviews verbatim, and no repeat interviews were necessary.

### Data analysis

Data were analysed by the same researcher using thematic analysis as described by Braun and Clarke.^17^ Coding and interpretation were conducted using NVivo12 software and occurred simultaneously with data collection, thus informing later interviews. Themes were developed when it was felt they encapsulated a central concept that was sufficiently important and salient both to the participant and the research question. As this iterative process progressed, findings were assessed for data saturation and the need for further recruitment and data collection was considered. Despite being offered copies of interview transcripts and initial analysis findings, participants did not request them.

## Results

Ten parents participated in nine interviews (Table 1). Ages of their children with asthma ranged between 2 and 18 years (Table 2). There was a wide range in severity of asthma, as well as time since diagnosis for those interviewed.

**Table 1.** Characteristics of study participants (*n* = 10)

Characteristic	Number of participants (%)
Sex	
Female	8 (80)
Male	2 (20)
Age, years	
30–39	4 (40)
40–49	6 (60)
Ethnicity	
Asian or Asian British	2 (20)
White	8 (80)
Civil status	
Single	2 (20)
Married or civil partnership	8 (80)
How participants heard about the project	
Social media	8 (80)
Schools	2 (20)

**Table 2.** Ages of children with an asthma diagnosis whose parents were interviewed (*n* = 12)

Age, years	Number of children (%)
0–5	3 (25)
6–11	6 (50)
12–18	3 (25)

Six themes were identified, each alluding to an aspect of parents' lived experience containing barriers and facilitators to receiving adequate asthma support.

### Theme 1: A power struggle with asthma

The initial process of asthma becoming part of parents’ lives was often described as being beyond their power to influence, thus forcing them to accept the limitations imposed by the disease. Factors pertaining to the condition itself, such as its severity, specific triggers and symptoms, as well as the context into which it enters, for instance, the child’s age and personality, and the family’s socioeconomic background, were considered to determine the extent to which the condition’s impact is felt:

*'I can only describe it as being traumatic. I've got a child that can't breathe 90% of the time.* [He] *asks me "What does it feel like to be able to breathe?"* […] *There* [were] *countless times in hospital when they said "This boy is not gonna make it". I never knew if I was coming home with my child. It was ... one of the most horrific times that I've ever endured*.' (P4)

However, as families learn about asthma and come to terms with its constraints, a new balance is struck. Despite being unable to eliminate asthma and its limitations on their day-to-day lives, parents learn how to contain it, managing its effects and mitigating its risks by adopting new behaviours, attitudes, and routines:

*'And it was just amazing to know how to deal with* [the asthma]. *And as the weeks have gone on and I'm more confident with dealing with it, I'm, like, really proud. And it’s so reassuring when he has a couple of days of coughing, and then we get over it because of the strategies we're using.'* (P8)

### Theme 2: Challenges in reaching a diagnosis

Reaching a diagnosis constituted a turning point for all participants as it enabled the start of appropriate medical treatment and gave parents a framework in which to manage the emotions caused by having an unwell child. Obtaining a diagnosis was often seen by parents as an unnecessarily drawn-out process, particularly when the child’s symptoms were milder. Common challenges were the perceived unwillingness of healthcare professionals to consider an asthma diagnosis, compounded by a disregard for parents’ concerns. Particularly with children aged <5 years, parents often found they had to press for a diagnosis to be made and the resistance they experienced left them feeling unsupported during this turbulent process:

*'… it was also very frustrating going backwards and forwards to the doctor’s. But we got there in the end. I just wish it hadn’t taken as long.'* (P3)

*'… not being able to do anything other than just comfort* [our daughter] *and wait for the doctors to see her is quite scary. So, actually getting the diagnosis and being able to request that brown inhaler, which we knew* [...] *worked, erm, it was a relief, more than anything.'* (P6)

Pre-diagnosis was an extremely challenging phase for some, marked by uncertainty, disruption caused by frequent school absences or trips to the health service, and a sense of being unequipped to care for their child:

*'Right from the beginning, understanding why* [my son’s] *got asthma would have been good.* [...] *So you’re prepared mentally, almost, that yeah, your child probably will be admitted to hospital at some point for an asthmatic episode.* [...] *Some clear explanation around the annual checks, how repeat prescription would work …*' (P5)

Some felt that the diagnosis process needed to be faster and less dependent on parents’ prompts, and that more information should be made available for children aged <5 years, or those awaiting a formal diagnosis. Parents whose child started displaying symptoms during the COVID-19 pandemic often commented on the uncertainty this brought about:

*'So there needs to be a better system around diagnosing asthma in the under-fives. Because what is in place at the moment is non-existent. And it’s appalling.*' (P8)

One parent recalled a phone conversation to an emergency department during a lockdown: *'And I said "I just want someone to see* [my son]*. I just want someone to see him and if it’s Covid, then I’m really sorry it’s Covid, but, I really don’t think it is*."' (P1)

### Theme 3: Partnership with healthcare services

Despite being foundational in the management of children’s asthma, the dynamic established between parents and the healthcare services was often fraught. Health professionals’ reluctance to take parents’ concerns seriously and acknowledge their expertise were seen as considerable barriers:

*'As a parent you’ve got that responsibility for your children anyway. But you do feel like you have to, put your foot down a bit and say, "No! You have to listen to me! I know my children!" And, to be honest, I don’t think a lot of GPs do*.' (P3)

Other issues raised were healthcare professionals’ tendency to adopt a reactive approach to asthma, minimise its severity, and fail to interpret and manage the condition within the family’s context. The health system itself was felt to be fractured and difficult to access:

*'They were just reading from a script, and no one really cared that I aged ten years, or* [my husband] *aged ten years, and that it was putting huge stress on the family. There was just no understanding of that at all*.' (P8)

*'And it’s only serious when it gets too serious.* [My daughter will] *have asthma for the rest of her life. Today she might have her inhaler three or four times if she’s having a bad day. Doesn’t mean she needs urgent care. Doesn't mean she’s gonna be poorly tomorrow. Just means she struggled a bit today. And I think that’s why it’s not seen as an emergency. But if she did have a full-blown asthma attack the prevention wouldn't have been there to stop that.'* (P10)

*'I have to keep going in the surgery and dropping the* [prescription request] *in the box, but obviously, 'cause I work, I mean it is open till six, but everyone’s racing one another, it’s quite hard to keep getting there to do that.*' (P1)

Facilitators to good asthma management included continuity of care, proactively contacting parents to check on asthma management, appointment and prescription reminders, and long-term one-to-one asthma support from an asthma nurse:

*'I know children who have diabetes have diabetes nurses, don’t they? And you’re attached to them, and they have their numbers, and they can text them and stuff if they’ve got problems, or parents just have that one contact.* [... ]*When* [children] *get older* [they] *don’t always want to speak to* [their] *parents, do* [they]*?* [They] *need someone* [they] *can go to outside.*' (P1)

### Theme 4: The importance of schools in managing asthma

Schools were recognised as key players and, when proactively taking steps to support children with asthma, had the potential to facilitate better asthma management. Most parents had a generally positive experience with how schools managed their child’s asthma but when this was not the case, it was a cause of great anxiety:

*'Five days a week, for six hours a day he was in school. He wasn't in my care, I’ve trusted him to other people. So I really, really needed them to hear me and to acknowledge what I was saying and follow through with the plan* [...].' (P4)

Lack of direct access to inhalers while in school was seen as the main issue. Some parents were also concerned about the following: deviations from management plans; inadequate asthma training of school staff; a disregard for the condition’s severity; poor communication around inhaler usage during school hours; and the fact that inhalers were given to other children who had not brought their own. Most parents had never met a school nurse to discuss their child’s asthma:

*'*[It] *would have been easier if* [my son] *just had the inhaler with him. And he would have easily taken it, 100%, if he felt a bit wheezy whilst playing sport. Rather than, kind of, hoping that he’ll tear himself away from something he’s enjoying with friends to ask a teacher to get inhaler, which might take up most of the break time.*' (P5)

*'*[My daughter] *knows where she should go if she has an asthma attack. She knows not to panic, she knows what’s gonna happen, she knows where her* [bag with the inhalers] *is* [...] *But I'm not overly convinced that they’d necessarily be sitting there if she got there. I think it would be someone else’s inhaler she ended up using. You know, it’d be nice if schools just had communal inhalers as a spare, just in case.*' (P10)

### Theme 5: A quest for knowledge

Parents often struggled to access timely, relevant information. Despite being identified as a primary and authoritative source, education provided by health professionals was often considered to be insufficient, directed solely at parents, and often lacking in advice on the practical aspects of asthma management. Education around inhaler technique was recognised as foundational and, when adequately performed, could be a pivotal moment in achieving better symptom control:

*'It’s just felt like a fight, constantly, to learn, instead of somebody taking control who is the expert in the subject!*' (P7)

'[My daughter’s] *asthma was really poorly controlled at that time* [...] *But then we saw the asthma nurse and we revised all the techniques with her and everything, and it’s settled. I didn't need to see the specialist.*' (P2)

Other sources of information such as online literature (namely NHS and Asthma UK websites), although sometimes useful were not always felt to be reliable. Lack of information was identified not only as an issue for parents, but also for the public:

*'There isn't that much online, really. If you go and look you read the same thing over and over again, but it doesn't actually tell you anything.*' (P10)

*'And because people aren't educated enough on* [asthma]*, people don't really take it seriously.* [...] *And I think if I didn't go through what I've gone through with my son, maybe I too would be just as ignorant.*' (P4)

### Theme 6: Lack of social network support

Despite social network support being recognised as important, most parents in the sample only received it from their immediate family. Lack of connections with other parents whose children had asthma or support groups were identified as barriers to good asthma management:

*'We don't have a massive support network. But those who do support us, have had to support us lots. If I had had every single day* [my son] *was off this year ill, if I had had that off work, I would no longer have a job* [...] *So we've had to rely on* [family and friends’] *support.'* (P8)

*'Yeah, it would be good to have more* [peer support]. *It’s reassuring, if anything, to hear people who've got the same experiences and reassuring to know that you're doing the right thing, because that’s what they're doing.*' (P9)

Several parents mentioned feeling that the public had a poor understanding of asthma and was, therefore, not able to offer support:

*'Whether it’s mild or severe,* [asthma’s] *so common, but yet a lot of people don't know anything about it.* [...] *What do you do if somebody’s having an asthma attack when you walk past them on the street? Nobody actually knows!* [...] *I only know it because I've got a poorly child! That should be something that everybody knows!'* (P4)

## Discussion

### Summary

While parents’ experiences vary, common barriers and facilitators to achieving good childhood asthma management are evident. Having a child with asthma had considerable impacts on parents’ mental health and wellbeing, and caused a sense of helplessness. The diagnosis process was marked by exceptional informational and emotional needs, which most often went unmet. This was compounded by a protracted diagnosis process, which is over-reliant on parents’ prompting and inflexible towards young children.

By dismissing parents’ concerns, failing to acknowledge their expertise, and adopting a reactive approach to symptom control, healthcare professionals were felt to be raising barriers. Lack of coordination and communication within the healthcare system was identified as an aggravating factor, leading to unnecessary delays in asthma detection and management.

Despite having the potential to support parents and enable good asthma management, interactions with schools and healthcare services were often a source of frustration and distress. Most parents were confident in their child’s ability to manage their symptoms but felt school policies hindered them. Timely, reliable, and relevant information, although foundational to adequate asthma management, was seen as difficult to access. Notably for this study sample, support from their wider social network was generally lacking. Parents interviewed did not describe a lack of finance or transport, or deprivation as a barrier to accessing basic asthma care.

### Strengths and limitations

A strength of this study was the diversity of experiences and sociodemographic backgrounds of the sample, which were representative of the study setting and allowed for the richness of the data collected. This was made possible by employing recruitment strategies targeting the relevant population. Similarities in this study’s results and those published in the literature offer confidence in its validity. Meanwhile, results that appear to be unique to this project’s context, such as the lack of social network support, give it relevance and make useful recommendations for improving clinical care in children with asthma.

Limitations of this study include challenges in recruitment that led to a small sample size. Unsuccessful snowballing points toward the lack of connections parents experience with other parents of children with asthma. While recruitment allowed for the relevant population to be targeted, it is likely to have caused a bias towards parents with high levels of involvement in their child’s asthma care and greater engagement with the healthcare system. A more comprehensive recruitment strategy, which could have been accessed by those with limited IT literacy and by non-English speakers, would have provided deeper insight into the experiences of those who may also struggle to access healthcare services. Furthermore, this study provides a one-sided perspective on this issue, is likely affected by recall bias, and is skewed toward participants who encountered hurdles when accessing asthma support in the past.

### Comparison with existing literature

There is no gold standard test for diagnosis of asthma. Difficulties in the diagnosis process resonate with findings from several studies highlighting the uncertainty,^18^ frustration,^9^ and personal toll^19^ of the pre-diagnosis phase, and the relief following diagnosis.^20^ Timely education at the point of diagnosis has previously been identified as a facilitator towards better management.^8,21,22^ Unlike other UK projects,^23–26^ this study pointed out that parents perceived lack of communication between health services as a cause for mismanagement and diagnosis delay. Participants also raised health professionals’ unwillingness to diagnose young children with asthma as a substantial barrier. Diagnosis in children aged <5 years is difficult^27,28^ and literature available on the experiences of parents of this specific age group in the UK is limited. A 2019 study in a UK primary care setting described how GPs and nurses found it hard to differentiate between asthma and other similarly presenting conditions at the extremes of age, thus making asthma diagnosis in children particularly challenging.^29^

Health professionals’ reluctance in taking the concerns of parents of children with asthma seriously has been described elsewhere.^19,30,31^ Non-UK studies have also identified parents’ issues in having their intimate knowledge of their child’s condition acknowledged,^32^ as well as the tendency for primary care to review asthma patients opportunistically or only when unwell.^29^ Difficulties in accessing health services raised by this study’s participants revolved mainly around the inconvenience and impracticality of the appointment and prescription systems. These barriers have been identified as one of the causes for over-reliance on the emergency services, particularly in vulnerable groups with less understanding of how to navigate the health system.^24^ Similarly to other studies,^32,33^ parents expressed a desire for their children to be involved in the decision-making process, and felt that being reviewed by a health professional who already knew their child improved the quality of care received.

Limited information is available on the impact of the UK school system on children’s asthma management. In keeping with findings from non-UK settings,^34,35^ school policies limiting accessibility of children’s inhalers were seen as barriers to adequate management.

Unlike findings from other studies,^20,36^ participants in this project did not identify their wider social network as a source of knowledge on the condition and its management. In fact, they were often the ones relaying information to others in their social sphere. Parents in this study expressed frustration at the lack of asthma awareness and education of the public, which they felt also impacted their child’s asthma management. These findings are corroborated by a qualitative analysis that found that adolescents in the UK felt the public was ignorant of asthma and inhaler use, particularly regarding mild and moderate cases.^37^ Several studies have pointed out the benefits of networking in asthma support groups, such as provision of information, practical tips, emotional support, and material assistance.^20,31,38^ They are also seen as a source of normative guidance, as parents compare their experience with those of people in a similar situation.^39^ Surprisingly, in this study, few parents were acquainted with others whose children also had asthma, and the reasons for this are unclear.

### Implications for research and practice

This study highlights the need for health professionals and policymakers to coproduce interventions or service provisions with carers and patients. Asthma diagnosis in children has been recognised as particularly problematic, and action is needed to improve the process, as well as the coordination and sharing of information between different parts of the health system, namely, the emergency department and primary care services. There is also need for increased targeted clinical support for parents whose child is awaiting formal diagnosis or is aged <5 years and likely to have asthma. This is important as uncertainty in diagnosis can lead to suboptimal adherence to prescribed treatments.^23^ Additionally, it is essential to enhance health professionals’ knowledge of parents’ needs, including awareness of the impact of asthma on family life, the indispensability of timely education, as well as the need for discussion of day-to-day aspects of asthma management. Availability of local asthma nurses as a single point of contact for children and their families for non-urgent issues would likely prove very beneficial to childhood asthma management.

Further research is needed to better understand the dynamics of the UK school environment on childhood asthma management. Areas requiring consideration include communication between schools and parents regarding asthma management, staff training in supporting children with asthma, and the impact of school policies on children’s access to inhalers.

At a local level, this study points to the benefits of facilitating the establishment of local support networks for children with asthma and their parents, possibly through partnerships between the local authority and primary care services.

Permeating all areas is the need for more accessible and relevant information, not only for children with asthma and their parents, but also their families, school staff, and the public.

Asthma affects millions of children, families, the health systems supporting them, and wider society. By exploring the views of parents, this study has shed light on barriers and facilitators to receiving childhood asthma support in an urban, multi-ethnic setting, with high levels of socioeconomic deprivation. Further research, employing different recruitment methods, is needed to better understand the views of parents with lower levels of engagement with the healthcare service and their child’s asthma care, and whether this patient cohort experiences different barriers and health outcomes. Lack of informational and emotional support in the stages leading up to asthma diagnosis was highlighted as an important challenge, particularly for parents whose child’s asthma symptoms start at an early age. Furthermore, an inflexible health system that is unaware of parents' needs poses a significant barrier in achieving adequate childhood asthma management. Qualitative research such as this can inform development and improvement of policy and services at national and local levels, with the potential for impacting children and their parents’ experience of managing a long-term condition.

## Data

The dataset relied on in this article is available from the corresponding author on reasonable request.
